# Exercise-Induced Excessive Blood Pressure Elevation Is Associated with Cardiac Dysfunction in Male Patients with Essential Hypertension

**DOI:** 10.1155/2022/8910453

**Published:** 2022-11-28

**Authors:** Binfeng Xia, Pengyu Cao, Li Zhang, Huihui Huang, Rongyu Li, Xia Yin

**Affiliations:** The Cardiovascular Center, First Hospital of Jilin University, 71 Xinmin Road, Changchun 130021, Jilin, China

## Abstract

**Objective:**

Cardiopulmonary exercise testing (CPET) has been used to explore the blood pressure response and potential cardiovascular system structure and dysfunction in male patients with essential hypertension during exercise, to provide a scientific basis for safe and effective exercise rehabilitation and improvement of prognosis.

**Methods:**

A total of 100 male patients with essential hypertension (aged 18–60) who were admitted to the outpatient department of the Center for Diagnosis and Treatment of Cardiovascular Diseases of Jilin University from September 2018 to January 2021 were enrolled in this study. The patients had normal cardiac structure in resting state without clinical manifestations of heart failure or systematic regularization of treatment at the time of admission. Symptom-restricted CPET was performed and blood pressure was measured during and after exercise. According to Framingham criteria, male systolic blood pressure (SBP) ≥210 mmHg during exercise was defined as exercise hypertension (EH), and the subjects were divided into EH group (*n* = 47) and non-EH group (*n* = 53). Based on whether the oxygen pulse (VO_2_/HR) plateau appeared immediately after anaerobic threshold (AT), the EH group was further divided into the VO_2_/HR plateau immediately after AT (EH-ATP) group (*n* = 19) and EH-non-ATP group (*n* = 28). The basic clinical data and related parameters, key CPET indicators, were compared between groups.

**Result:**

Body mass index (BMI) visceral fat, resting SBP, and SBP variability in EH group were significantly higher than those in non-EH group. Moreover, VO_2_/HR at AT and the ratio of VO_2_/HR plateau appearing immediately after AT in EH group were significantly higher than those in the non-EH group. The resting SBP, 15-minute SBP variability, and the presence of VO_2_/HR plateau were independent risk factors for EH. In addition, work rate (WR) at AT but also WR, oxygen consumption per minute (VO_2_), VO_2_/kg, and VO_2_/HR at peak were significantly lower in the EH-ATP group compared to the EH-non-ATP group. Peak diastolic blood pressure (DBP) increment and decreased △VO_2_/△WR for AT to peak were independent risk factors for VO_2_/HR plateau appearing immediately after AT in EH patients.

**Conclusion:**

EH patients have impaired autonomic nervous function and are prone to exercise-induced cardiac dysfunction. EH patients with exercise-induced cardiac dysfunction have reduced peak cardiac output and exercise tolerance and impaired vascular diastolic function. CPET examination should be performed on EH patients and EH patients with exercise-induced cardiac dysfunction to develop precise drug therapy and effective individual exercise prescription, to avoid arteriosclerosis and exercise-induced cardiac damage. The retrospective study protocol was approved by medical ethics committee of the First Hospital of Jilin University (AF-IRB-032-06 No. 2021-015). The study was registered with the Chinese Clinical Trials Register, registration number: ChiCTR2100053140.

## 1. Introduction

Hypertension is one of the most important global health challenges and a leading risk factor of cardiovascular diseases (CVDs). Studies have shown that in 2015, the number of hypertensive patients was about 1.13 billion worldwide, among which the age-standardized prevalence rate of males and females was 24% and 20%, respectively [1]. It is predicted that in 2025, the global number of adult hypertension will increase to 1.56 billion [2], and each year about 17 million people die from CVD, among whom high blood pressure-related complications contribute to 9 million deaths [3]. Therefore, it is particularly important to detect hypertension and target organ damage early and treat them actively by not only medical therapy but also exercise among the obese patients to reduce blood pressure [4].

At present, three measurements of resting state blood pressure (systolic blood pressure ≥140 mmHg and/or diastolic blood pressure ≥90 mmHg) in different days are still used to diagnose hypertension and evaluate hypertension according to resting blood pressure [4]. However, with the development of exercise load test (treadmill, power bike, etc.) technology, the understanding of blood pressure is not limited to resting blood pressure level. Change of blood pressure during exercise, one of the hot spots in the field of cardiovascular research, has attracted the attention of the majority of researchers. The concept and diagnostic criteria of exaggerated blood pressure response to exercise (EBPRE) were first proposed by Dlin et al. in 1983 [5], referring to the phenomenon of abnormally increased blood pressure during and/or after exercise under a certain exercise load, also known as exercise hypertension (EH). Recent studies have found that patients with EH may have potential structural changes and functional disorders of cardiovascular system [6] which are also major risk factors for increased incidence of cardiovascular and cerebrovascular events and mortality [7, 8]. Currently, there is no unified standard for the diagnosis of EH. According to the Framingham standard, EH is systolic blood pressure (SBP) ≥210 mmHg in males or SBP ≥190 mmHg in females during exercise [9, 10].

Cardiopulmonary exercise testing (CPET) is the most accurate method for detecting cardiopulmonary function during exercise and is the gold standard for evaluating exercise tolerance and cardiopulmonary fitness [11, 12]. CPET can monitor not only abnormal blood pressure response during exercise but also abnormal changes in electrocardiogram, cardiac output, cardiac stroke volume, and aerobic endurance, to detect early or potential cardiac dysfunction. In addition to electrocardiogram and exercise blood pressure monitoring, it also measures respiratory gas exchange during exercise [13]. We hypothesized that the potential cardiac dysfunction in EH patients may be associated with reduced cardiac functional reserve and reduced vascular diastolic function. In this retrospective study, male patients with essential hypertension were selected to explore whether there is early or potential cardiac dysfunction in EH patients and whether the cardiac dysfunction is associated with cardiopulmonary fitness in response to exercise.

## 2. Methods

### 2.1. Study Population and Group

This retrospective study included a total of 386 18–60-year-old male patients with essential hypertension admitted to outpatient department of cardiovascular disease of the First Hospital of Jilin University from September 2018 to January 2021. Patients with abnormal cardiac structure (105) and patients who disagreed to undergo CPET (181) were excluded from this study. The normal cardiac structure was defined by echocardiographic measurement from M-mode echocardiogram in the parasternal long-axis view as follows: (1) left atrium anteroposterior diameter is 19–39 mm, (2) left ventricular end diastolic diameter is 35–55 mm, (3) interventricular septal depth is 7–11 mm, (4) left ventricular posterior wall thickness is 7–11 mm, (5) left ventricular ejection fraction is ≥50% in resting state, and (6) left ventricular mass index ≤115 g/m^2^. The retrospective study protocol was approved by medical ethics committee of the First Hospital of Jilin University (AF-IRB-032-06 No. 2021-015). The study was registered with the Chinese Clinical Trials Register, registration number: ChiCTR2100053140.

The diagnostic criteria for essential hypertension were in line with the guidelines for hypertension prevention and Treatment in China in 2018 [4]: (1) hypertension can be diagnosed if systolic blood pressure ≥140 mmHg and/or diastolic blood pressure ≥90 mmHg 3 times on different days without anti-hypertensive drugs and (2) the patient had a history of hypertension and was taking anti-hypertensive drugs, even though the blood pressure was <140/90 mmHg at the time of measurement. All subjects were excluded from the following conditions: (1) secondary hypertension; (2) history of coronary heart disease or exercise testing with clear evidence of myocardial ischemia; (3) various types of cardiomyopathy and valvular heart disease; (4) clinical symptoms and signs of heart failure and/or echocardiography indicating abnormal cardiac function; (5) abnormal lung function caused by the history of lung diseases; (6) anemia, thyroid dysfunction, stroke, severe liver and kidney insufficiency, and other diseases in the acute stage; (7) not suitable for exercise due to other reasons; and (8) use of *β*-blockers.

According to the Framingham standard [9, 10], there were 47 participants in the EH group and 53 participants in the non-EH group. Subsequently, according to the CPET, the EH group was further divided into two subgroups based on whether the VO_2_/HR plateau appeared immediately after anaerobic threshold (AT), namely, the VO_2_/HR plateau immediately after AT (EH-ATP) group (*n* = 19) and non-VO_2_/HR plateau after AT (EH-non-ATP) group (*n* = 28).

### 2.2. Data Collection

Baseline characteristics, including age and biochemical data such as total cholesterol (TC), triglyceride (TG), high-density lipoprotein cholesterol (HDL-C), low-density lipoprotein cholesterol (LDL-C), uric acid, and creatinine, were obtained from electronic medical records. Before CPET, all patients completed the blood biochemical tests by venous blood sampling under fasting state in the morning.

### 2.3. Arterial Stiffness

Subjects were placed supine on an examination bed and rested for 5 minutes. Left and right ankle brachial index (ABI) and left and right brachial-ankle pulse wave velocity (baPWV) were measured by an arteriosclerosis detection device (BP-203RPEIII, Omron Dalian Company Limited, China).

### 2.4. Determination of Body Compositions

Subjects took off their shoes, socks, and heavy clothing and stood on a body composition analyzer (SECA mBCA515, seca GmbH, Germany) for body composition measurement, body mass index (BMI), and visceral fat content.

### 2.5. CPET

Cardiopulmonary function detector (QPFT/CPET/FX, Cosmed Srl, Italy) was used to detect the changes of oxygen consumption (VO_2_) and carbon dioxide (CO_2_) emission at resting state, AT state, and peak state by increasing 20 watts/min. At the same time, heart rate and blood pressure were monitored by 12-lead electrocardiogram recorder and dynamic blood pressure monitor. The CPET parameters including oxygen consumption per kilogram body weight (VO_2_/kg), heart rate (HR), oxygen pulse (VO_2_/HR), work rate (WR) at resting, AT, and peak states, and blood pressure at resting state and 1 and 3 minutes after exercise were recorded. The exercise test was terminated if the patient developed any of the following subjective or objective conditions: abnormal hemodynamic or ECG exercise response or other causes such as dyspnea, angina, or lower extremity muscle fatigue [11, 12].

### 2.6. Measurement of Blood Pressure

Resting blood pressure, peak blood pressure, and blood pressure 1 and 3 minutes after exercise were obtained from CPET. Blood pressure at 5, 7, 10, and 15 minutes after exercise was measured by a calibrated arm electronic blood pressure monitor (Omron HEM-7200, Omron Dalian Company Limited, China). The variability of blood pressure within 15 minutes was calculated from the blood pressure values at 1, 3, 5, 7, 10, and 15 minutes after exercise.

### 2.7. Statistical Analysis

SPSS 23.0 software was used for statistical analysis. The measurement data conforming to normal distribution were described by mean ± standard deviation, and non-normally distributed variables were presented as medians (interquartile range, IQR). Categorical variables were expressed as numbers and percentages. Continuous variables between the groups were compared with means of one-way analysis of variance or Mann–Whitney *U* test; chi-square test was carried out for dichotomous variables. In all analyses, a two-tailed *P* < 0.05 was considered statistically significant. To minimize potential bias, corrections were performed by multifactor binary logistic regression, in which the indices and variables showing *P* < 0.05 in the univariate analyses were introduced and were used to distinguish independent influencing factors. The results are presented as odds ratios (ORs) with 95% confidence intervals (95% CIs).

## 3. Results

### 3.1. Comparison of General Clinical Data between EH Group and Non-EH Group

**Figure 1:**
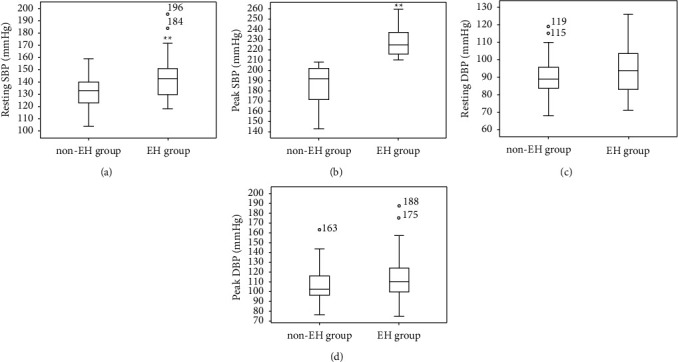
Comparison of resting and peak blood pressure between EH group and non-EH group.

There were no significant differences in age, TC, TG, HDL-C, LDL-C, baPWV, ABI, resting diastolic blood pressure (DBP), peak DBP increase, and DBP variability within 15 minutes after exercise between the two groups (Table 1). BMI (30.04 ± 3.47 vs. 28.05 ± 3.66 kg/m^2^, *P*=0.006), visceral fat (4.36 ± 1.28 vs. 3.70 ± 1.07 L, *P*=0.006), resting SBP (142.70 ± 17.10 vs. 131.32 ± 12.93 mmHg,  *P* < 0.01), peak SBP increase (86 vs. 55 mmHg, <0.01), and SBP variability (24.02 vs. 14.20 mmHg, <0.01) within 15 minutes after exercise in the EH group were significantly higher than those in the non-EH group (Table 1, Figure 1).

### 3.2. Comparison of CPET Parameters between EH Group and Non-EH Group

**Figure 2:**
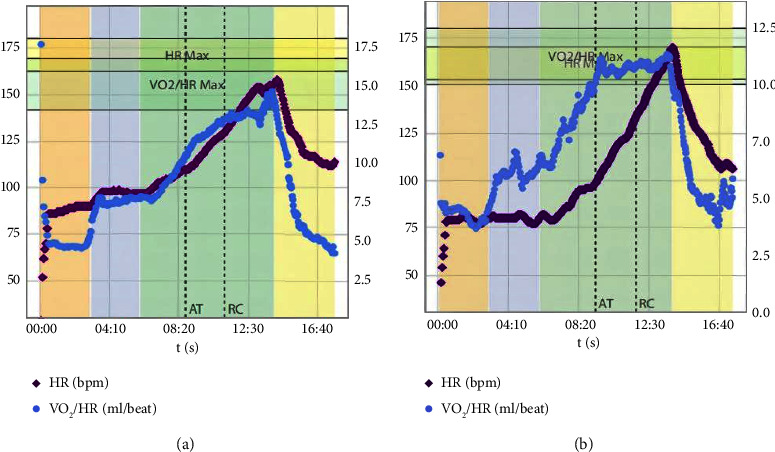
The characteristic examples for VO_2_/HR plateau and non-plateau.

There were no differences in CPET parameters at resting and peak states between the two groups, except that the VO_2_/HR was higher at AT state in EH group than in non-EH group (9.96 ± 1.71 vs. 9.10 ± 1.71 ml/beat, *P*=0.02, Table 2). The ratio of VO_2_/HR plateau immediately after AT in EH group was significantly higher than that in non-EH group (40.43 vs. 20.75%, *P*=0.03, Table 2, Figure 2).

### 3.3. Multivariate Binary Logistic Regression Analysis of Influence of the Occurrence of EH

The factors with statistical significance in univariate analysis were included as independent variables, and the occurrence of EH during exercise was taken as the dependent variable for multivariate binary logistic regression analysis. The results showed that resting SBP (OR = 1.08, 95% CI: 1.03–1.12, *P* < 0.01, Table 3), 15-minute SBP variability (OR = 1.16, 95% CI: 1.08–1.26, *P* < 0.01, Table 3), and VO_2_/HR plateau (OR = 4.16, 95% CI: 1.16–14.85, *P*=0.03, Table 3) after AT are independent risk factors for EH.

### 3.4. Comparison of Clinical Data between EH-ATP Group and EH-Non-ATP Group

As shown in Table 4, there were no significant differences in age, TC, TG, HDL-C, LDL-C, uric acid, creatinine, baPWV and ABI, visceral fat, BMI, resting blood pressure, peak SBP increase, and blood pressure variability within 15 minutes after exercise between the two groups. The increase of peak DBP in EH-ATP group was significantly higher than that in EH-non-ATP group (27.84 ± 13.04 vs. 24.04 ± 11.18 mmHg, *P* < 0.01).

### 3.5. Comparison of CPET Parameters between EH-ATP Group and EH-Non-ATP Group

CPET parameters in resting state showed no difference between EH-ATP and EH-non-ATP groups (Table 5). At peak state, VO_2_ (1.63 vs. 1.89 L/min, *P*=0.04), VO_2_/kg (18 vs. 21 ml/kg/min, *P*=0.02), VO_2_/HR (10.76 ± 1.70 vs. 12.36 ± 2.14 ml/beat, *P* < 0.01), and WR (136.53 ± 24.07 vs. 153.54 ± 28.32 watt, *P*=0.04) in the EH-ATP group were lower than those in EH-non-ATP group (Table 5). At AT state, only WR in the EH-ATP group was lower than that in the EH-non-ATP group (70.05 ± 17.42 vs. 84.32 ± 24.78 watt, *P*=0.04, Table 5). Furthermore, △VO_2_/△WR for AT to peak was significantly decreased in the EH-ATP group (7.37 vs. 9.01 ml/Watt, *P* < 0.01, Table 5).

### 3.6. Multivariate Binary Logistic Regression Analysis of Influence of the Occurrence of EH-ATP

The factors with statistical significance in univariate analysis were taken into consideration as independent variables, and the occurrence of EH-ATP during exercise was taken as the dependent variable for multivariate binary logistic regression analysis. The results showed that peak DBP increment (OR = 1.06, 95% CI: 1.01–1.12, *P*=0.03, Table 6) and decreased △VO_2_/△WR for AT to peak (OR = 0.57, 95% CI: 0.37–0.88, *P*=0.01, Table 6) are independent risk factors for VO_2_/HR plateau appearing immediately after AT in EH patients.

### 3.7. Comparison of Cardiopulmonary Fitness, Cardiac Stroke Volume, and Heart Rate Response

**Figure 3:**
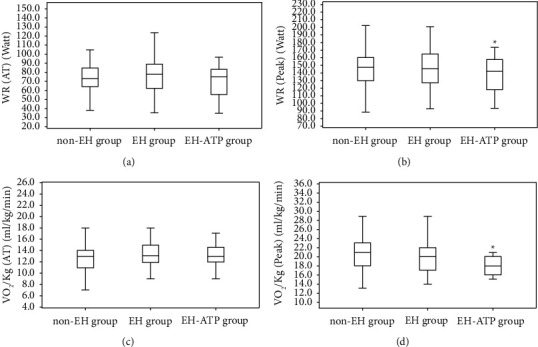
Comparison of cardiopulmonary fitness during exercise.

**Figure 4:**
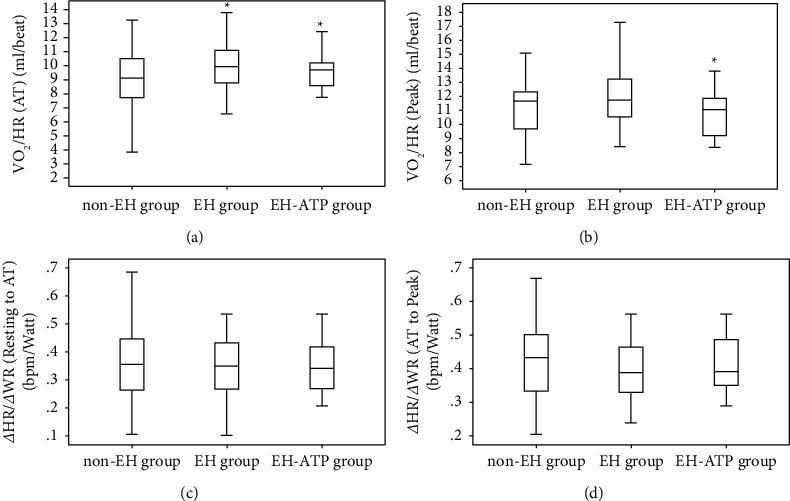
Comparison of cardiac stroke volume and heart rate response during exercise.

The cardiopulmonary fitness of WR (136.53 ± 24.07 vs. 143.92 ± 27.88 watt) and VO_2_/kg (18 vs. 21 ml/kg/min) were both significantly lower in EH-ATP group than in non-EH group only at peak state but not at AT state (*P* < 0.05, Figure 3). The cardiac stroke volume of VO_2_/HR was significantly higher at AT state (9.64 ± 1.43 vs. 9.10 ± 1.71 ml/beat), but lower at peak state (10.76 ± 1.70 vs. 11.24 ± 2.11 ml/beat) in the EH-ATP group than in the non-EH group (*P* < 0.05, Figures 4(a) and 4(b)). No significant difference was found in heart rate response among the non-EH group, EH group, and EH-ATP group not only from resting to AT but also from AT to peak (Figures 4(c) and 4(d)).

## 4. Discussion

The current study is to explore the correlation between EH and cardiovascular dysfunction. Our study showed that EH patients have higher BMI, visceral fat content, and resting SBP in resting state, and the rate of exercise-induced cardiac dysfunction is higher than that in non-EH patients. Multivariate binary logistic regression analysis showed that resting SBP, 15-minute SBP variability, and the presence of VO_2_/HR plateau are independent risk factors for EH. We found that EH patients with exercise-induced cardiac dysfunction have a significant decrease in both exercise tolerance and oxygen utilization efficiency. Moreover, we showed that peak DBP increment and decreased △VO_2_/△WR for AT to peak are independent risk factors for VO_2_/HR plateau appearing immediately after AT in EH patients.

The presence of EH may indicate a subhealth state, and for hypertensive patients, the presence of EH may increase the damage of target organs and the occurrence of cardiovascular and cerebrovascular events. Therefore, early detection of EH in hypertensive patients is particularly important. Studies have shown that the occurrence of EH may be related to hyperactivity of sympathetic nervous system [14], excessive activation of RAAS system [15], vascular endothelial dysfunction, arteriosclerosis [10], and inflammatory response [16]. Furthermore, obesity and increased visceral fat have been proved to accelerate the process of atherosclerosis and are associated with the induction of EH [17]. Consistent with these findings, we found that higher BMI and increased visceral fat are more likely to induce EH in hypertensive patients. Previous studies showed that increased visceral fat can accelerate the process of atherosclerosis possibly due to insulin resistance [18]. Visceral fat is thought to release fatty acids into the portal vein, leading to insulin resistance in the liver and muscles [19]. Subsequently, insulin resistance mediates the development of atherosclerosis through activating the sympathetic nervous system and decreasing the bio-activity of nitric oxide [19]. In addition, other studies have shown that central obesity may increase arterial stiffness by inducing smooth muscle cell proliferation, vascular cell calcification, and oxidative stress [20]. It may also accelerate arterial stiffness in increased arterial intimal media thickness and decreased arterial lumen diameter via changes in endovascular inflammatory factors and endothelial function [21]. At rest, ABI and baPWV are the main methods to evaluate the degree of vascular blockage in lower limbs and arteriosclerosis. ABI < 0.9 is considered as the critical value of suspected lower limb vascular blockage, and baPWV value increases with the severity of atherosclerosis [22]. Blood pressure variability represents the degree of blood pressure fluctuation in a certain period of time. It exists independently of blood pressure level and can reflect autonomic nervous function, so the abnormal blood pressure variability is a risk factor for worsening atherosclerosis [23]. In this study, ABI and baPWV of EH patients did not change significantly; the resting SBP and the variability of SBP within 15 minutes after exercise were significantly increased. Multivariate binary logistic regression analysis showed that resting SBP, 15-minute SBP variability, and the presence of VO_2_/HR plateau are independent risk factors for EH. Our results revealed no significant difference in the degree of arteriosclerosis at the resting state but higher autonomic nervous dysfunction during exercise in EH patients compared to non-EH patients. Miyai et al. [14] found that sympathetic activity in EH patients is over-activated. This will induce increase in norepinephrine levels during exercise, which leads to increased cardiac output and peripheral resistance and decreased arterial compliance, resulting in excessive increase in SBP during exercise. Taken together, obesity and increased visceral fat lead to abnormal SBP variability in hypertensive patients during and after exercise through the over-activation of sympathetic nerve, causing the occurrence of EH. Therefore, for hypertension patients, lifestyle intervention should be carried out, to control the risk factors of atherosclerosis such as BMI and visceral fat. On the other hand, it is important that exercise treatment should be used for the obese patients to reduce blood pressure values and improve the activity of sympathetic nerve for preventing the occurrence of EH.

During exercise, with the increase of exercise intensity, the demand for oxygen of skeletal muscle increases, and the left ventricle increases cardiac output to meet the oxygen demand of muscle [24]. The increase of cardiac output is mainly achieved through the increase of stroke volume and heart rate. Before AT state, the increase of cardiac output mainly depends on the increase of stroke volume, and stroke volume generally reaches its peak at exercise intensity near the AT state [13, 24]. After AT state, the increase of cardiac output mainly depends on the increase of heart rate. If the VO_2_/HR does not rise with the increase of exercise intensity after the AT state, it indicates a decrease of cardiac stroke volume [13], which is an important manifestation of exercise-induced cardiac dysfunction. In our study, the VO_2_/HR level of EH patients increased significantly at AT state, but there was no difference in heart rate and WR, indicating that in EH patients, the after-loading of left ventricle (SBP) is greater during moderate intensity exercise, and the heart needs to do more work to meet the body's need for oxygen. Recent studies have reported that the elevation of SBP during moderate exercise is a better predictor of left ventricular hypertrophy (LVH) than resting SBP in hypertensive patients [25, 26]. As maximal exercise is effort-dependent, the measured peak SBP sometimes does not reflect the exact SBP elevation in maximal exercise due to the poor fitness level [26]. Excessive elevation of SBP during moderate exercise can be used to identify hypertensive patients with high risk of developing LVH [26]. In addition, we also found that the presence of VO_2_/HR plateau in moderate exercise is higher in the EH group and it is an independent risk factor for EH, which may be a vital sign of early decrease in cardiac function due to excessive elevation of blood pressure.

VO_2_/kg is an important indicator of exercise tolerance and is used to evaluate the level of aerobic capacity [11, 12]. In 2016, aerobic capacity was defined as the fifth vital sign, which is closely related to the occurrence and prognosis of cardiovascular diseases [27]. Our study found that the peak VO_2_, VO_2_/kg, WR, and VO_2_/HR are significantly reduced in EH patients with VO_2_/HR plateau immediately after AT state during exercise. These indicate that both exercise tolerance and maximum stroke volume are significantly reduced in these patients, which is an important sign of early decline in exercise-induced cardiac function. At AT state, VO_2_/kg and HR in EH-ATP group did not change, but WR decreased significantly than that in non-EH-ATP group. Furthermore, the parameter of oxygen utilization efficiency, △VO_2_/△WR for AT to peak, decreased significantly in the EH-ATP group. It was an independent risk factor for VO_2_/HR plateau in EH patients, suggesting the decrease in aerobic work efficiency of muscle above moderate intensity exercise. This may be a more important cause of the decline in exercise tolerance except for the decrease in peak VO_2_. Previous studies have shown that decreased type I muscle fiber in patients with chronic heart failure induces the reduction in the number of skeletal muscle capillaries and the number of oxidase, resulting in a decline in the utilization efficiency of oxygen [28]. In the EH-ATP group, the heart rate response after AT state did not increase significantly, indicating that the hyperactive sympathetic nerve cannot compensate for the decline of stroke volume by increasing heart rate, leading to the decrease of peak cardiac output, which is the potential reason for the decline of exercise tolerance. Moreover, the peak DBP increase during exercise was significantly increased in the EH-ATP group and it was an independent risk factor for VO_2_/HR plateau appearing immediately after AT in EH patients, indicating exercise-induced cardiac dysfunction, which may be related to the exercise-induced vascular diastolic dysfunction. Taken together, EH patients with decreased exercise-induced stroke volume may have a potential decline in the aerobic work efficiency of skeletal muscle and impaired vascular diastolic function, which may contribute to the development of LVH. Thus, special attention should be paid to the safety and effectiveness of exercise intensity to avoid the exercise-induced cardiovascular injury in EH-ATP patients. In addition, no significant difference was found in cardiopulmonary fitness of EH-ATP patients before AT exercise intensity, which is consistent with the previous studies showing that regular moderate intensity aerobic exercise treatment is safe and effective for hypertension patients [29, 30]. A meta-review of 33 systematic reviews has shown that high-intensity interval training (HIIT) can effectively improve the cardiopulmonary fitness, exercise tolerance, and muscle structure [31]. It seems that exercise-induced diastolic dysfunction does not impair PH at rest; however, it can contribute to an abnormally exaggerated pulmonary artery pressure in response to exercise with elevated pulmonary capillary wedge pressure [32]. Therefore, when EH-ATP patients perform effective high-intensity intermittent exercise [33], the time of high-intensity exercise should be controlled to avoid exaggerated intra-cardiac pressure and the development of LVH. Our findings suggest that EH patients should not only lower resting BP but also improve exercise-induced VO_2_/HR plateau (decreased stroke volume) to prevent the development of LVH.

In conclusion, compared to non-EH patients, EH patients have worse autonomic nervous function and are more prone to exercise-induced cardiac dysfunction. EH patients with exercise-induced cardiac dysfunction have decreased peak cardiac output and exercise tolerance and impaired vascular diastolic function. Therefore, exercise with an intensity above the AT state for a long time may aggravate damage of the heart. In view of this, it is recommended to conduct early screening of CPET for patients with essential hypertension to detect potential exercise-induced cardiac dysfunction in EH patients as early as possible. We can achieve safe and effective anti-hypertension through precise drug therapy and individualized exercise treatment to inhibit the process of arteriosclerosis and improve the long-term prognosis. Future studies are needed to determine the optimal individual level of exercise to achieve aerobic performance and avoid the onset of EH in hypertensive patients.

### 4.1. Limitation

Firstly, the patients included in this study were all outpatients from the department of cardiology at the First Hospital of Jilin University, only representing the single center study. Secondly, the sample size of patients included in this study was limited, and the results of the study need to be confirmed by a larger sample size. Thirdly, the subjects were all patients with essential hypertension and were not compared to the general healthy population. Fourthly, although EH patients with decreased exercise-induced stroke volume may be related to the exercise-induced vascular diastolic dysfunction, endothelial function was not evaluated in the present study. Future studies are required to assess endothelial function, such as a flow-mediated vasodilation and SBP at moderate exercise, to clarify the influence of vascular endothelial dysfunction on exercise-induced cardiac dysfunction in EH patients. Fifthly, EH patients with decreased exercise-induced stroke volume may have a potential decline in the aerobic work efficiency of skeletal muscle above moderate exercise. To achieve safe and effective anti-hypertension through individualized exercise treatment, the intensity and duration of HIIT should be determined in the future studies. Finally, female subjects should be studied to investigate whether there is any gender difference.

## Figures and Tables

**Table 1:** Comparison of clinical data between EH group and non-EH group.

	EH group (*n* = 47)	Non-EH group (*n* = 53)	*P* value
Age (year)	40.77 ± 10.70	39.81 ± 9.20	0.63
TG (mmol/L), median (IQR)	2.11 (1.37, 3.22)	1.95 (1.31, 2.52)	0.49
TC (mmol/L), median (IQR)	4.91 (4.10, 5.31)	4.86 (4.31, 5.76)	0.45
LDL (mmol/L)	1.20 ± 0.20	1.24 ± 0.31	0.51
HDL (mmol/L)	3.16 ± 0.77	3.07 ± 0.86	0.57
Uric acid (mmol/L)	444.26 ± 87.78	423.89 ± 94.86	0.27
Creatinine (mmol/L)	81.00 ± 17.45	78.88 ± 15.21	0.53
Left bapwv (cm/s), median (IQR)	1535 (1380, 1685)	1490 (1327, 1624)	0.22
Right bapwv (cm/s), median (IQR)	1531 (1371, 1909)	1471 (1334, 1616)	0.22
Left ABI (ratio)	1.12 ± 0.07	1.12 ± 0.10	0.99
Right ABI (ratio)	1.12 ± 0.08	1.12 ± 0.09	0.79
Visceral fat (L)	4.36 ± 1.28	3.70 ± 1.07	<0.01^*∗∗*^
BMI (kg/m^2^)	30.04 ± 3.47	28.05 ± 3.66	<0.01^*∗∗*^
Resting SBP (mmHg)	142.70 ± 17.10	131.32 ± 12.93	<0.01^*∗∗*^
Resting DBP (mmHg)	95.23 ± 13.92	90.32 ± 11.03	0.05
Peak SBP increase (mmHg), median (IQR)	86 (72, 95)	55 (38, 70)	<0.01^*∗∗*^
Peak DBP increase (mmHg), median (IQR)	15 (9, 26)	14 (8, 25)	0.732
15 min SBP variability (mmHg), median (IQR)	24.02 (16.06, 28.25)	14.20 (10.69, 20.91)	<0.01^*∗∗*^
15 min DBP variability (mmHg), median (IQR)	7.00 (4.31, 10.79)	5.35 (3.46, 8.76)	0.08

TC: total cholesterol, TG: total triglycerides, HDL-C: high-density lipoprotein cholesterol, LDL-C: low-density lipoprotein cholesterol, BMI: body mass index, ABI: ankle brachial index, baPWV: brachial-ankle pulse wave velocity, SBP: systolic blood pressure, and DBP: diastolic blood pressure. ^*∗*^*P* < 0.05; ^*∗∗*^*P* < 0.01.

**Table 2:** Comparison of CPET parameters between EH group and non-EH group.

	EH group (*n* = 47)	Non-EH group (*n* = 53)	*P* value
Resting			
VO_2_ (L/min), median (IQR)	0.37 (0.31, 0.40)	0.36 (0.32, 0.41)	0.97
VO_2_/kg (ml/kg/min), median (IQR)	4 (4, 5)	4 (4, 5)	0.09
HR (bpm)	89.66 ± 11.09	89.70 ± 12.78	0.99
VO_2_/HR (ml/beat), median (IQR)	3.98 (3.49, 4.55)	4.03 (3.61, 4.33)	0.94
AT			
VO_2_ (L/min), median (IQR)	1.14 (0.91, 1.34)	1.03 (0.91, 1.21)	0.07
VO_2_/kg (ml/kg/min), median (IQR)	13 (12, 15)	13 (11, 15)	0.61
HR (bpm), median (IQR)	115 (108, 124)	116 (108, 127)	0.80
VO_2_/HR (ml/beat)	9.96 ± 1.71	9.10 ± 1.71	0.02^*∗*^
WR (watt)	78.55 ± 23.01	74.49 ± 16.29	0.31
Peak			
VO_2_ (L/min), median (IQR)	1.67 (1.42, 2.03)	1.64 (1.48, 1.94)	0.41
VO_2_/kg (ml/kg/min), median (IQR)	20 (17, 22)	21 (18, 23)	0.29
HR (bpm)	148.45 ± 15.79	150.49 ± 18.67	0.31
VO_2_/HR (ml/beat)	11.71 ± 2.11	11.24 ± 2.11	0.25
WR (watt)	146.66 ± 27.76	143.92 ± 27.88	0.59
Resting to AT			
△HR/△WR (bpm/watt)	0.35 ± 0.10	0.36 ± 0.12	0.59
AT to peak			
△HR/△WR (bpm/watt)	0.45 ± 0.11	0.47 ± 0.12	0.73
Resting to peak			
△HR/△WR (bpm/watt)	0.40 ± 0.09	0.42 ± 0.11	0.32
VO_2_/HR plateau [*n* (%)]	19 (40.43)	11 (20.75)	0.03^*∗*^

VO_2_: oxygen consumption per minute, VO_2_/kg: oxygen consumption per kilogram of body weight per minute, HR: heart rate per minute, VO_2_/HR: oxygen pulse, WR: work rate, and △HR/△WR: heart rate increment per unit work rate. ^*∗*^*P* < 0.05; ^*∗∗*^*P* < 0.01.

**Table 3:** Binary logistic regression analysis of EH in hypertensive patients during exercise.

	OR	95% CI	*P* value
VO_2_/HR plateau (*n*)	4.16	1.16–14.85	0.03^*∗*^
BMI (kg/m^2)^	1.07	0.83–1.38	0.62
Visceral fat (L)	1.39	0.72–2.69	0.33
Resting SBP (mmHg)	1.08	1.03–1.12	<0.01^*∗∗*^
15 min SBP variability (mmHg)	1.16	1.08–1.26	<0.01^*∗∗*^
VO_2_/HR (AT) (ml/beat)	1.36	0.95–1.96	0.09

SBP: systolic blood pressure and VO_2_/HR (AT): VO_2_/HR at anaerobic threshold. ^*∗*^*P* < 0.05; ^*∗∗*^*P* < 0.01.

**Table 4:** Comparison of clinical data between EH-ATP group and EH-non-ATP group.

	EH-ATP group (*n* = 19)	EH-non-ATP group (*n* = 28)	*P* value
Age (year)	42.37 ± 10.58	39.68 ± 10.84	0.41
TG (mmol/L), median (IQR)	1.57 (1.31, 2.14)	2.38 (1.57, 3.38)	0.15
TC (mmol/L)	4.53 ± 0.46	5.01 ± 1.23	0.12
LDL (mmol/L)	1.19 ± 0.18	1.22 ± 0.21	0.61
HDL (mmol/L), median (IQR)	2.84 (2.401, 3.34)	3.19 (2.77, 3.79)	0.12
Uric acid (mmol/L), median (IQR)	455 (358, 528)	457 (391, 488)	0.72
Creatinine (mmol/L), median (IQR)	76.6 (71.21, 87.83)	81.15 (72.48, 95.38)	0.29
Left bapwv (cm/s), median (IQR)	1550 (1445, 1793)	1550 (1338, 1585)	0.07
Right bapwv (cm/s), median (IQR)	1543 (1423, 1790)	1511 (1354, 1624)	0.11
Left ABI (ratio)	1.13 ± 0.07	1.11 ± 0.07	0.42
Right ABI (ratio)	1.11 ± 0.07	1.13 ± 0.08	0.46
Visceral fat (L), median (IQR)	4.3 (3.0, 5.3)	4.0 (3.3, 5.28)	0.98
BMI (kg/m^2^)	29.22 ± 3.75	30.60 ± 3.21	0.18
Resting SBP (mmHg)	144.79 ± 18.89	141.29 ± 15.97	0.49
Resting DBP (mmHg)	95.79 ± 12.14	94.86 ± 15.22	0.83
Peak SBP increase (mmHg)	82.79 ± 25.31	87.57 ± 20.04	0.47
Peak DBP increase (mmHg)	27.84 ± 13.04	24.04 ± 11.18	<0.01^*∗∗*^
15 min SBP variability (mmHg), median (IQR)	23.55 (16.06, 27.68)	25.44 (15.60, 28.80)	0.54
15 min DBP variability (mmHg), median (IQR)	7.94 (4.39, 13.38)	6.47 (3.09, 9.67)	0.07

TC: total cholesterol, TG: total triglycerides, HDL-C: high-density lipoprotein cholesterol, LDL-C: low-density lipoprotein cholesterol, BMI: body mass index, ABI: ankle brachial index, baPWV: brachial-ankle pulse wave velocity, SBP: systolic blood pressure, and DBP: diastolic blood pressure. ^*∗*^*P* < 0.05; ^*∗∗*^*P* < 0.01.

**Table 5:** Comparison of CPET parameters between EH-ATP group and EH-non-ATP group.

	EH-ATP group (*n* = 19)	EH-non-ATP group (*n* = 28)	*P* value
Resting			
VO_2_ (L/min), median (IQR)	0.37 (0.31, 0.40)	0.36 (0.31, 0.40)	0.67
VO_2_/kg (ml/kg/min), median (IQR)	4 (4, 5)	4 (4, 4)	0.14
HR (bpm)	90.42 ± 11.89	89.14 ± 10.07	0.70
VO_2_/HR (ml/beat), median (IQR)	3.98 (3.49, 4.55)	4.03 (3.61, 4.33)	0.94
AT			
VO_2_ (L/min), median (IQR)	1.07 (0.91, 1.26)	1.16 (0.96, 1.45)	0.31
VO_2_/kg (ml/kg/min), median (IQR)	13 (12, 15)	13 (11.25, 15.75)	0.79
HR (bpm)	114.58 ± 11.19	118.68 ± 15.39	0.33
VO_2_/HR (ml/beat)	9.64 ± 1.43	10.18 ± 1.87	0.29
WR (watt)	70.05 ± 17.42	84.32 ± 24.78	0.04^*∗*^
Peak			
VO_2_ (L/min), median (IQR)	1.63 (1.39, 1.88)	1.89 (1.49, 2.16)	0.04^*∗*^
VO_2_/kg (ml/kg/min), median (IQR)	18 (16, 20)	21 (18, 22)	0.02^*∗*^
HR (bpm)	147.32 ± 13.31	149.21 ± 17.47	0.69
VO_2_/HR (ml/beat)	10.76 ± 1.70	12.36 ± 2.14	<0.01^*∗∗*^
WR (watt)	136.53 ± 24.07	153.54 ± 28.32	0.04^*∗*^
Resting to AT			
△VO_2_/△WR (ml/watt), median (IQR)	10.58 (9.12, 12.41)	10.07 (8.49, 11.27)	0.32
△HR/△WR (bpm/watt)	0.35 ± 0.10	0.35 ± 0.11	0.96
AT to peak			
△VO_2_/△WR (ml/watt), median (IQR)	7.37 (5.96, 8.95)	9.01 (7.89, 10.24)	<0.01^*∗∗*^
△HR/△WR (bpm/watt)	0.50 ± 0.12	0.45 ± 0.13	0.22
Resting to peak			
△VO_2_/△WR (ml/watt), median (IQR)	8.70 (7.88, 9.94)	9.44 (8.54, 10.85)	0.12
△HR/△WR (bpm/watt)	0.42 ± 0.09	0.39 ± 0.09	0.38

VO_2_: oxygen consumption per minute, VO_2_/kg: oxygen consumption per kilogram of body weight per minute, HR: heart rate per minute, VO_2_/HR: oxygen pulse, WR: work rate, △VO_2_/△WR: oxygen consumption increment per unit work rate, and △HR/△WR: heart rate increment per unit work rate. ^*∗*^*P* < 0.05; ^*∗∗*^*P* < 0.01.

**Table 6:** Binary logistic regression analysis of EH-ATP in hypertensive patients during exercise.

	OR	95% CI	*P* value
Model 1			
Peak DBP increase (mmHg)	1.07	1.01–1.14	0.02^*∗*^
VO_2_ (peak) (L/min)	1.77	0.02–158.39	0.80
VO_2_/kg (peak) (ml/kg)	0.99	0.73–1.33	0.92
VO_2_/HR (peak) (ml/beat)	0.54	0.26–1.12	0.10
Model 2			
Peak DBP increase (mmHg)	1.05	1.01–1.10	0.02^*∗*^
WR (AT) (watt)	0.98	0.94–1.02	0.25
WR (peak) (watt)	0.98	0.95–1.01	0.25
Model 3			
Peak DBP increase (mmHg)	1.06	1.01–1.12	0.03^*∗*^
△VO_2_/△WR (ml/watt)	0.57	0.37–0.88	0.01^*∗*^
(AT to peak)			

DBP: diastolic blood pressure, VO_2_ (peak): oxygen consumption per minute at peak, VO_2_/kg (peak): oxygen consumption per kilogram of body weight per minute at peak, VO_2_/HR (peak): oxygen pulse at peak, WR (AT): work rate at anaerobic threshold, WR (peak): work rate at peak, and △VO_2_/△WR (AT to peak): oxygen consumption increment per unit work rate from AT to peak. ^*∗*^*P* < 0.05; ^*∗∗*^*P* < 0.01.

## Data Availability

The data and materials used to support the findings of the study are available from the corresponding author upon request.
